# Validation of the Adherence Barriers Questionnaire – an instrument for identifying potential risk factors associated with medication-related non-adherence

**DOI:** 10.1186/s12913-015-0809-0

**Published:** 2015-04-10

**Authors:** Sabrina Müller, Thomas Kohlmann, Thomas Wilke

**Affiliations:** 1 Institut für Pharmakoökonomie und Arzneimittellogistik (IPAM), Hochschule Wismar, Philipp-Müller-Straße 12, Wismar, 23966 Germany; 2 Institut für Community Medicine, Universitätsmedizin Greifswald, Greifswald, Germany

**Keywords:** Adherence, Compliance, Persistence, Reasons for Non-adherence/Non-persistence, Barriers of adherence/persistence

## Abstract

**Background:**

Medication non-adherence is a major challenge in the real-life treatment of chronically ill patients. To meet this challenge, adherence interventions with a tailored approach towards patient-specific adherence barriers that are identified with a reliable and practicable questionnaire are needed. The aim of this investigation was to develop and validate such a questionnaire, the “Adherence Barriers Questionnaire (ABQ)”.

**Methods:**

The German ABQ was developed and tested in 432 patients with atrial fibrillation in a multicentre observational cohort study. Evaluation of the questionnaire included an assessment of internal consistency as well as factor analysis. Criterion-related external validity was assessed by comparing the ABQ score with (1) the degree of self-reported adherence and (2) the time in therapeutic range which describes the anticoagulation quality achieved by patients treated with oral anticoagulation.

**Results:**

The final 14-item ABQ scale demonstrated high internal consistency (Cronbach’s alpha = 0.820). Factor analysis identified a three-factor solution, representing intentional adherence barriers with 5 items (31.9% of the variance), medication-/health care system-related adherence barriers with 5 items (13.3% of the variance) and unintentional adherence barriers with 4 items (7.7% of the variance).

The ABQ correlated significantly with self-reported non-adherence (Spearman’s rho = 0.438, p < 0.001) as well as time in therapeutic range (Spearman’s rho = − 0.161, p < 0.010). Patients with above-average ABQ scores (increased number and/or strength of existing adherence barriers) were significantly (p < 0.005, Pearson Chi-Square) more likely to have a poor anticoagulation quality (TTR < 60%) than patients with a lower ABQ score (44.6% versus 27.3%).

**Conclusions:**

The ABQ is a practicable, reliable and valid instrument for identifying patient-specific barriers to medication-related adherence. Future research is required to examine the ability of the ABQ to identify patient perception/behaviour changes over time which may be important for the measurement of success of adherence interventions.

**Electronic supplementary material:**

The online version of this article (doi:10.1186/s12913-015-0809-0) contains supplementary material, which is available to authorized users.

## Background

Appropriate medication adherence, which can be defined as the extent to which a patient’s drug-taking behaviour corresponds with agreed instructions from a health care provider [1-3], is essential for realising the potential health benefits of a certain medication-based treatment [1-5]. Many patients, especially those with chronic diseases, experience difficulties in adhering to a recommended treatment plan, and medication non-adherence (NA) with average rates of affected patients of about 30-50% is a major challenge in the real-life treatment of those patients [3-5].

To meet this challenge and improve patient outcomes, it is important to develop both effective and practical interventions for enhancing medication adherence. In the last years, a high number of scientific publications confirmed the need for improving medication-related adherence [2,6-8]. But there is an obvious lack of efficacy of existing adherence interventions/programs [2,9,10], especially with regards to improvements of long-term adherence and of associated clinical outcomes [11]. Available evidence shows that there are multiple reasons for this lack of efficacy. One of these reasons may be the inability of most of the interventions/programs to customise adherence interventions on patient-specific needs and preferences [3]. Moreover, existing research regarding factors causing medication-related NA, which we will call adherence barriers, shows that there is a variety of explanations for that phenomenon, and that these different factors explain medication-related NA in specific patients to a completely different extent. So, recent research proposes to differentiate, at a minimum, between intentional and unintentional NA [3,12-14]. Similarly to this, the World Health Organization (WHO) described NA as being a complex and multidimensional construct, which is related to socio-economic factors, health care system-related, and disease- and therapy-specific as well as patient-related factors [5]. So, to ensure efficacy of adherence interventions a tailored approach towards patient-specific adherence barriers is needed. Consequently, lack of knowledge with regards to the importance of specific adherence barriers in a specific patient leads to a lack of efficacy of adherence interventions.

If adherence interventions/programs need to consist of patient-specific barrier-reducing measures [5-9], a reliable and practical tool for identification of those barriers is needed.

In the past, some adherence self-report instruments, which assessed both the degree of non-adherence as well as reasons of observed non-adherence, have been developed. One example is the Morisky Medication Adherence Scale (MMAS) [15], which has shown suitability in verification of NA but generates limited information about the predictors influencing NA. Moreover, it hardly covers all of the known adherence barriers. Consequently, it has been validated as instrument assessing the degree of non-adherence only. Other instruments, like the Brief Medication Questionnaire or the Beliefs about Medicines Questionnaire [16,17], only addresses specific domains of barriers (e.g. patient beliefs). Furthermore, there are questionnaires addressing different categories of barriers in a more detailed manner, but these questionnaires are generally disease-specific instruments [18,19] and, therefore, of limited suitability for use in general clinical practice. Correspondingly, a review analysing 43 different adherence scales showed that, so far, there has been less focus on exploring patient-specific barriers and how information may be useful in supporting wise medicine use [20].

The aim of this study was, therefore, to develop a questionnaire, called “Adherence Barriers Questionnaire (ABQ)”, which measures (1) whether any adherence barriers are present in a patient, and (2) to which of the most important categories these barriers belong.

## Methods

### Item development and ABQ questionnaire

By conducting a systematic review of the relevant literature in the years 2008–2010 (see Additional file 1), the most frequent observable factors associated with medication related NA were identified; an updated review after data analysis that had been done revealed that no additional general adherence barriers were discussed in recent years (see Additional file 2). Generally, in accordance with existing literature, the identified adherence barriers could be classified into four main groups:

- Medication-related barriers: Most frequently observed adherence barriers were the complexity of medication regimes [21-23] and fear of/experience with side-effects [24,25].
- Health care system-related barriers: In this category, the direct/indirect medication-related costs patients have to bear (co-payments, waiting times, long journeys to reach the doctor, etc.) as well as a poor patient-physician relationship were the most frequently observed barriers [26-29].
- Patient-related unintentional barriers: Factors associated with unintentional NA are those such as depression [30,31], dementia, or the level of forgetfulness or the degree of carefulness [31-34].
- Patient-related intentional barriers: Existing research shows that intentional barriers may be the most important single adherence barrier [3,12,33]; general attitudes towards the treatment, the health-care system and medication or health beliefs [17,35], as well as coping behaviour [36-38] are the most important influencing elements of intentional adherence.

The results of this review were assessed by a scientific team consisting of all authors and formed the basis for item construction. A total of 16 items addressing all described adherence barriers were considered for the initial version of the ABQ. Each item was formulated as a statement. With regards to the response structure, a rating scale was chosen by assessing the grade of information exploitation and, on the other hand, the risk of overtaxing respondents. Finally, a 4-point Likert scale was defined, which deliberately left out a mean response option to force the respondents to a decision. The possible answers were “strongly agree”, “generally agree”, “generally disagree”, and “strongly disagree”, which were given values from 1 to 4 or rather 4 to 1 depending on the formulation of each item (a higher score indicated a higher influence of a certain barrier on patient’s perceptions). Based on patients’ responses, a general as well as item- and subscale-specific ABQ scores indicating the number and strength of adherence barriers present in a patient were calculated.

In general, patients’ self-reports always face the risk that the information provided by respondents may be distorted, even with a pre-defined response scale. In particular, social desirability bias could be a problem in terms of adherence-related questions. To control for the tendency of respondents to answer in a manner that will be viewed favourably by health care providers (or others), we added five more questions at the end of the interview. For this purpose, three items of the Social Desirability Scale-17 [39] were adopted and supplemented by two other health-related items. The items were also formulated as statements and could be answered either with “yes” or “no”. All items were aligned so that the answer “yes” indicated a stronger socially desirable response bias. These additional items were not part of the ABQ, but were used for assessment of the validity of the ABQ in subgroups classified by high/low social desirability response bias.

### Survey

We applied the ABQ in a multicentre, non-interventional, prospective observational cohort study addressing the general treatment of patients with atrial fibrillation (AF) in general practices in Germany (ACT-AF study). Written informed consent was obtained from each patient in this study and the study protocol was approved by an independent ethical committee. AF is the most common significant cardiac rhythm disorder that is associated with substantial lethality from stroke and thromboembolism. According to current guidelines, a treatment with oral anticoagulation is recommended for AF patients with a high risk of stroke [40]. Non-adherence to oral anticoagulation (OAC) seems to be a considerable problem and leads to an increase in adverse medical events, including stroke and bleeding events [41].

In the ACT-AF study, 71 participating general practitioners (GPs) were asked to include AF patients (ICD10 code I48; no further criteria) who were at least 18 years old and not participating in any other study (first patient in: May 2009, last patient out: May 2011). The data documented in the study included the clinical and sociodemographic background of each patient as well as anticoagulation treatment information and all international normalised ratio (INR) values related to anticoagulation therapy of the patients in the prospective observational period of 12 months; new oral anticoagulants were not available at the time of the study so that anticoagulation was based on vitamin-K-antagonists (VKA) only. Additional data were collected via written questionnaires at the end of the study period; this last survey took place on average 290.6 days after study inclusion. In our analysis, we included only patients who participated in this last survey; for the statistical analyses, only data regarding patients who completed the ABQ without any data gaps were used.

The developed ABQ was applied in its written form in a patient survey at study sites. Furthermore, during this survey, patients were asked to fill out a self-report instrument that measured the extent of potential NA (modified “Adherence to Refills and Medications Scale – ARMS” [42]). The ARMS is a validated instrument (one of the few questionnaires which were validated using at least two different external criteria and, among them, at least one was a clinical outcome). Furthermore, the ARMS contains hardly any questions which measure adherence barriers; in this respect, it focuses on assessing the level of non-adherence. Nevertheless, the original ARMS had to be reduced by two items that rather seemed to measure causes of NA (carelessness and cost) than the extent of NA. The modified ARMS consisted of 10 items (4-point Likert scales) with a total score from 10 to 40, wherein a higher score indicated higher non-adherence (see Additional file 3).

The ACT-AF study protocol, which included all mentioned questionnaires, was approved by the Ethics Commission of the University of Greifswald (Germany). The ABQ as well as all other questionnaires were applied in the German language.

### Questionnaire validation and statistical analysis

All analyses were conducted using SPSS Statistics 17.0 (2008 SPSS Inc.).

The internal consistency reliability of the ABQ was examined by calculating the Cronbach’s alpha, which describes the questionnaire’s homogeneity [43]. In general, an alpha ≥ 0.8 is desirable [43,44]. Simultaneously, the item-total correlation coefficient for the different items was evaluated, where a correlation ≥ 0.3 is seen as adequate [45].

The internal structure of the ABQ was evaluated by principal components exploratory factor analysis. This analysis generates possible subscales that are represented by sets of items within the questionnaire. The Kaiser-Meyer-Olkin measure of sampling adequacy and Bartlett’s test of sphericity were used to confirm the eligibility of the database for using this factor analysis. The initial number of factors was determined by using eigenvalues > 1 as well as scree plots examination. Sets of items generated by the Promax-rotated component matrix were assessed to define whether they fitted into the identified subdomains of the questionnaire. Items with a loading of > 0.4 were considered to adequately represent a factor.

The external validity of the questionnaire was investigated by using two different criteria. First, an assessment of the Spearman’s rho correlation of the ABQ score with the score of the used self-report adherence measure (modified ARMS) was done by determining the amount of correlation as a measure of the validity. In this analysis, only patients having completed the ABQ as well as the modified ARMS scale were included.

Second, a Spearman’s rho correlation of the ABQ scale with the available clinical outcome “time in therapeutic range” (TTR) was conducted. The TTR is a patient-specific assessment of the quality of oral anticoagulation therapy, and indicates the proportion of treatment time in which a patient’s INR value was in the pre-defined therapeutic range (INR between 2.0 and 3.0). The TTR is strongly correlated with the medication intake, and therefore, a good clinical indicator of medication-related adherence. In this analysis, only patients having completed the ABQ without any data gaps, having been prescribed VKA, and with two INR values available during the prospective observational period of the study, were included.

Furthermore, the proportion of patients with good anticoagulation quality among respondents with high versus low ABQ scores (above or below the median) was compared by using a chi-square test. According to current guidelines, a good anticoagulation quality can be defined as TTR ≥ 60% (at least 60% of the observed days are within the target range) [40]. The chi-square test should confirm the hypothesis that patients with a high number and/or above-average strength of existing adherence barriers are less likely to reach this level of TTR.

To consider a potential social desirability bias, all described analyses for assessing the criterion-related validity were repeated separately in patients without a high tendency to answer in a manner that will be viewed favourably by health care providers. This tendency was appraised by an additional scale gained from the last five questions addressing social desirability with a score ranging from 0 to 5. Patients with a score of five were defined as most probably biased regarding social desirability, and were excluded.

## Results

### Adherence Barriers Questionnaire (ABQ)

Our developed ABQ consisted of 16 different items. Five items referred to intentional adherence barriers (items 4, 5, 6, 7, 12), four items to unintentional adherence barriers (items 2, 9, 10, 13), four items to medication-related barriers (items 11, 14, 15a, 15b), and three items to health care system-related barriers (items 1, 3, 8).

### Sample characteristics

Of the 786 AF-patients registered in the ACT-AF study, 570 (72.5%) participated in the survey. However, 138 patients did not respond to all ABQ questions, so that 432 patients completed the ABQ questionnaire without any data gaps. These 432 patients who formed the basis of our analysis had a mean age of 72.7 years; 45.6% were female. These patients suffered from AF for 6.6 years on average, and took a mean number of 6.6 long-term medications as reported by the treating physician. Most frequent observed comorbidities were hypertension (83.8%) and diabetes (38.4%).

In the external validation analyses, two subsamples of the sample of 432 patients were analysed. In the first analysis (ABQ versus modified ARMS scale), 401 patients with completed responses to both the ABQ and the modified ARMS questionnaire were included. Of these patients 44.6% showed poor self-reported adherence (ARMS scores below the median of 11). In the second analysis, 371 patients with completed responses to the ABQ, having received vitamin K antagonists as anticoagulation treatment, and having at least two INR value measures available, were included. Table 1 shows the main characteristics of the different patient samples.

**Table 1:** **Characteristics of the patient samples**

Variables	All AF patients in the ACT-AF study		AF patients participating in the survey		AF patients having completed the ABQ questionnaire without any data gaps		AF patients having completed both the ABQ and the modified ARMS scale		AF patients with complete ABQ data, who received VKA treatment and with at least 2 INR values available	
N	786		570		432		401		371	
Average age in years	73.17	(SD: 9.24)	73.08	(SD: 9.12)	72.74	(SD: 9.34)	73.09	(SD: 8.73)	72.79	(SD: 8.92)
Female gender	360	(45.8%)	264	(46.3%)	197	(45.6%)	178	(44.4%)	162	(43.7%)
Ø CHA_2_DS_2_-VASc score^+^	3.76	(SD: 1.63)	3.86	(SD: 1.62)	3.84	(SD: 1.66)	3.89	(SD: 1.63)	3.82	(SD: 1.64)
Ø Duration since first AF diagnosis in years	6.25	(SD: 5.43)	6.59	(SD: 5.83)	6.59	(SD: 5.83)	6.80	(SD: 5.73)	6.60	(SD: 5.34)
Average number of prescribed long-term medications as reported by treating physicians	6.33	(SD: 2.65)	6.32	(SD: 2.62)	6.62	(SD: 5.55)	6.30	(SD: 2.58)	6.25	(SD: 2.55)
Living arrangements										
Living alone	240	(30.5%)	167	(29.3%)	125	(28.9%)	114	(28.4%)	105	(28.3%)
Living with a partner	530	(67.4%)	392	(68.8%)	299	(69.2%)	279	(69.6%)	260	(70.1%)
Living in a care home	16	(2.1%)	11	(1.9%)	8	(1.9%)	8	(2.0%)	6	(1.6%)
Education level										
University degree	58	(7.4%)	41	(7.2%)	28	(6.5%)	25	(6.2%)	24	(6.5%)
Apprenticeship	600	(76.3%)	440	(77.2%)	333	(77.1%)	312	(77.8%)	286	(77.1%)
Without apprenticeship	128	(16.3%)	89	(15.6%)	71	(16.4%)	64	(16.0%)	61	(16.4%)
Employment status										
Employed	58	(7.4%)	37	(6.5%)	31	(7.2%)	27	(6.7%)	29	(7.8%)
Unemployed	8	(1.0%)	9	(1.6%)	9	(2.1%)	8	(2.0%)	6	(1.6%)
Pensioner	710	(90.3%)	517	(90.7%)	385	(89.1%)	361	(90.0%)	329	(88.7%)
Other	10	(1.3%)	7	(1.2%)	7	(1.6%)	5	(1.2%)	7	(1.9%)
Cognitive impairment	65	(8.3%)	56	(9.8%)	39	(9.0%)	36	(9.0%)	31	(8.4%)
Hypertonia	655	(83.3%)	485	(85.1%)	362	(83.8%)	341	(85.0%)	314	(84.6%)
Diabetes mellitus type ½	274	(34.9%)	56	(37.9%)	166	(38.4%)	157	(39.2%)	147	(39.6%)
Dementia	54	(6.9%)	44	(7.7%)	34	(7.9%)	31	(7.7%)	26	(7.0%)
Depression	126	(16.0%)	98	(17.2%)	74	(17.1%)	65	(16.2%)	60	(16.2%)
Mental illness	63	(8.0%)	51	(8.9%)	40	(9.3%)	37	(9.2%)	31	(8.4%)
Cancer	84	(10.7%)	63	(11.1%)	46	(10.6%)	44	(11.0%)	43	(11.6%)

*+ stroke risk factors (C = “Congestive heart failure” - 1 score point; H = “Hypertension” - 1 score point; A = Age ≥ 75 -2 score points; D = “Diabetes mellitus” - 1 score point; S = “Stroke/TIA” - 2 score points; V = Vascular disease - 1 score point; A = Age: 65–74 - 1 score point; S = “Sex category: female” - 1 score point).*

AF: Atrial Fibrillation, ACT-AF: Ambulant Care and Treatment of Atrial Fibrillation, ABQ: Adherence Barriers Questionnaire, ARMS: Adherence to Refills and Medications scale, INR: International Normalized Ratio.

### ABQ distribution analysis

Each ABQ item was scored 1 to 4 with a higher score indicating a stronger influence/importance of the specific adherence barrier from a respondent’s point of view. Table 2 displays the distribution characteristics of the patients’ responses to the 16 ABQ items. Most of the items showed a right-skewed distribution of the scores and thus had similar effects on the answering pattern of the patients (particularly important with regards to the inter-item-correlation). The only exception was item 8 (“I feel that co-payments for medicines are a great burden”), with a skewness of −0.135. This indicates that it was difficult for patients to contradict this statement; which is also illustrated by the mean and median of the scores (item 8 showed the highest values with a mean of 2.66 and a median of 3.0).

**Table 2:** Distribution of responses to ABQ-items*

Item	Mean	Median	SD	Skewness
**Item 1:** “I fully understand what my doctor, nurse or the people at my pharmacy have explained to me so far”.^+^	1.58	1.00	0.700	1.082
**Item 2:** „I can mention the names of my medicines and their scope without hesitation”.^+^	2.33	2.00	0.975	0.149
**Item 3:** „I trust my doctor and agree to my therapy plan together with him”.^+^	1.28	1.00	0.512	1.846
**Item 4:** “My medications help me only if I take them absolutely regularly as recommended”.^+^	**1.27**	1.00	0.512	1.961
**Item 5:** „Medicines are all poisonous. You should avoid taking medicines at all if possible”.	1.82	2.00	0.902	0.849
**Item 6:** „I feel basically healthy. Therefore I am sometimes unsure whether I really have to take my medicines daily”.	1.79	2.00	0.869	0.971
**Item 7:** „I take my medicines every day automatically at a fixed time or on fixed occasions”.^+^	1.36	1.00	0.609	1.847
**Item 8:** „I feel that co-payments for medicines are a great burden”.	**2.66**	**3.00**	1.059	**−0.135**
**Item 9:** „I frequently forget things on an everyday basis”.	2.32	2.00	0.906	0.435
**Item 10:** „Generally I often feel bad, and sometimes I feel discouraged and depressed.”	2.24	2.00	0.908	0.329
**Item 11:** „I frequently have problems taking my medications or it is difficult for me to keep me on the accompanying conditions of the medication intake”.	1.73	1.50	0.902	1.184
**Item 12:** „I have to overcome obstacles to my healthcare”.	2.03	2.00	1.120	0.678
**Item 13:** „I really would need help on an everyday basis (and particularly related to my treatment with medicines). But I do not get any help”.	1.55	1.00	0.855	1.565
**Item 14:** „I am really frightened of the side effects of my medicines.”	1.91	2.00	0.854	0.797
**Item 15a:** „In case I already noticed or in case I would notice side effects related to my medicines: I have talked or would talk to my doctor about them as soon as possible”.^+^	1.28	1.00	0.605	**2.543**
**Item 15b:** „In case I already noticed or in case I would notice side effects related to my medicines: I have stopped/would stop my medications or took/would take less of them”.	1.80	1.50	0.976	1.005

*Questionnaire was applied in German; translation into English has been done by the authors; score per item 1–4; n = 432 patients/respondents.

^+^This item was reverse coded.

Bold numbers represent maximum/minimum values.

ABQ: Adherence Barriers Questionnaire, SD: Standard Deviation.

### Reliability

Cronbach’s α for the original ABQ-scale was 0.814 and demonstrated a good internal consistency. Item-total correlations for the 16 items ranged from 0.225 to 0.634 (Table 3). Statement 6 of the questionnaire (“I feel basically healthy. Therefore I am sometimes unsure whether I really have to take my medicines daily.”) showed the lowest item-scale correlation (0.225), and Cronbach’s α increased to 0.817 if this item was deleted. Furthermore, item 5 (“Medicines are all poisonous. You should avoid taking medicines at all if possible.”) demonstrated a low item-total correlation coefficient (0.263). Deleting this item led to an increased Cronbach’s α of 0.815. Additionally, statement 15a with an item-scale correlation coefficient of 0.274 was below the critical threshold of 0.3, but in this case, removing the item led to a decreased internal consistency (α = 0.812). Consequently, the ABQ questionnaire was reduced by removing items 5 and 6, and the examination of the internal consistence reliability was revised (Table 3). Item-total correlation in the reduced 14-item-ABQ scale ranged from 0.265 to 0.640, and upon removing items 5 and 6, the Cronbach’s α increased to 0.820.

**Table 3:** **Item-total correlations for the original and the reduced ABQ**

Item	Original 16-item ABQ		Reduced 14-item ABQ	
	(Cronbach’s α: 0.814)		(Cronbach’s α: 0.820)	
	Item-total correlation coefficient	Cronbach’s α if item is deleted	Item-total correlation coefficient	Cronbach’s α if item is deleted
**Item 1:** “I fully understand what my doctor, nurse or the people at my pharmacy have explained to me so far”.	0.599	0.795	0.634	0.798
**Item 2:** „I can mention the names of my medicines and their scope without hesitation”.	0.371	0.808	0.397	0.813
**Item 3:** „I trust my doctor and agree to my therapy plan together with him”.	0.461	0.805	0.443	0.811
**Item 4:** “My medications help me only if I take them absolutely regularly as recommended”.	0.394	0.808	0.375	0.814
**Item 5:** „Medicines are all poisonous. You should avoid taking medicines at all if possible”.	0.263	0.815	-	-
**Item 6:** „I feel basically healthy. Therefore I am sometimes unsure whether I really have to take my medicines daily”.	0.225	0.817	-	-
**Item 7:** „I take my medicines every day automatically at a fixed time or on fixed occasions”.	0.351	0.809	0.326	0.816
**Item 8:** „I feel that co-payments for medicines are a great burden”.	0.400	0.807	0.395	0.814
**Item 9:** „I frequently forget things on an everyday basis”.	0.416	0.804	0.431	0.81
**Item 10:** „Generally I often feel bad, and sometimes I feel discouraged and depressed”.	0.534	0.796	0.567	0.799
**Item 11:** „I frequently have problems taking my medications or it is difficult for me to keep me on the accompanying conditions of the medication intake”.	0.634	0.789	0.640	0.793
**Item 12:** „I have to overcome obstacles to my healthcare”.	0.505	0.798	0.546	0.801
**Item 13:** „I really would need help on an everyday basis (and particularly related to my treatment with medicines). But I do not get any help”.	0.600	0.792	0.610	0.796
**Item 14:** „I am really frightened of the side effects of my medicines”.	0.424	0.804	0.393	0.812
**Item 15a:** „In case I already noticed or in case I would notice side effects related to my medicines: I have talked or would talk to my doctor about them as soon as possible”.	0.274	0.812	0.265	0.819
**Item 15b:** „In case I already noticed or in case I would notice side effects related to my medicines: I have stopped/would stop my medications or took/would take less of them”.	0.373	0.808	0.337	0.818

ABQ: Adherence Barriers Questionnaire.

### Internal validity

During development of the questionnaire, four subscales were originally defined (medication-related adherence barriers, health care system-related barriers, and intentional as well as unintentional barriers). The corresponding items were initially assigned to these scales on the basis of their content. Factor analysis of the original ABQ-scale (16 items) based on eigenvalues also suggested a four-factor solution, which explained 55.72% of the variance. However, factor analysis based on the reduced ABQ-scale (without items 5 and 6) identified a three-factor solution (Table 4). This three-factor solution still explains more than 50% of the variance (52.88%), which supported the decision to reduce the original scale (parallel to the advantage in terms of ease of use for respondents). Factor 1 (representing intentional adherence barriers) demonstrated an eigenvalue of 4.459 and accounted for 31.9% of the variance. In whole, five items load on this factor (Table 4). The second component containing five items can be labelled as subscale describing medication- or health care system-related adherence barriers; it had an eigenvalue of 1.864 and explained 13.3% of the variance. Finally, items 1 and 2 as well as items 9 and 10 can be summarised as unintentional adherence barriers. These items showed a maximum loading on the third factor. Factor 3 had an eigenvalue of 1.080 and explained 7.7% of the variance.

**Table 4:** Factor analysis based on the reduced ABQ*

	Factor 1 (subscale of intentional NA risk) component loading	Factor 2 (subscale of medication-/health care system-related NA risk) component loading	Factor 3 (subscale of unintentional NA risk) component loading
**Eigenvalue**	4.459	1.864	1.080
**Variance explained**	31.850%	13.317%	7.716%
Item			
**Item 1:** “I fully understand what my doctor, nurse or the people at my pharmacy have explained to me so far”.			0.672
**Item 2:** „I can mention the names of my medicines and their scope without hesitation”.			0.678
**Item 3:** „I trust my doctor and agree to my therapy plan together with him”.	0.795		
**Item 4:** “My medications help me only if I take them absolutely regularly as recommended”.	0.775		
**Item 7:** „I take my medicines every day automatically at a fixed time or on fixed occasions”.	0.670		
**Item 8:** „I feel that co-payments for medicines are a great burden”.		0.602	
**Item 9:** „I frequently forget things on an everyday basis”.			0.749
**Item 10:** „Generally I often feel bad, and sometimes I feel discouraged and depressed”.			0.763
**Item 11:** „I frequently have problems taking my medications or it is difficult for me to keep me on the accompanying conditions of the medication intake”.		0.755	
**Item 12:** „I have to overcome obstacles to my healthcare”.		0.688	
**Item 13:** „I really would need help on an everyday basis (and particularly related to my treatment with medicines). But I do not get any help.”		0.796	
**Item 14:** „I am really frightened of the side effects of my medicines”.		0.677	
**Item 15a:** „In case I already noticed or in case I would notice side effects related to my medicines: I have talked or would talk to my doctor about them as soon as possible”.	0.659		
**Item 15b:** „In case I already noticed or in case I would notice side effects related to my medicines: I have stopped/would stop my medications or took/would take less of them”.	0.385		

*Extraction Method: Principal Component Analysis, Rotation Method: Promax.

ABQ: Adherence Barriers Questionnaire, NA: Non-Adherence.

For the first subscale (intentional adherence barriers), Cronbach’s α was 0.653 and the item-total correlations ranged from 0.298 to 0.539. A range from 0.407 to 0.641 with regards to the item-total correlation coefficients of the second subscale (medication- or health care system-related adherence barriers; α = 0.749) could be observed. The third subscale (unintentional adherence barriers) demonstrated a Cronbach’s α of 0.709 and a range of item-total correlations from 0.433 to 0.551.

**Figure 1:**
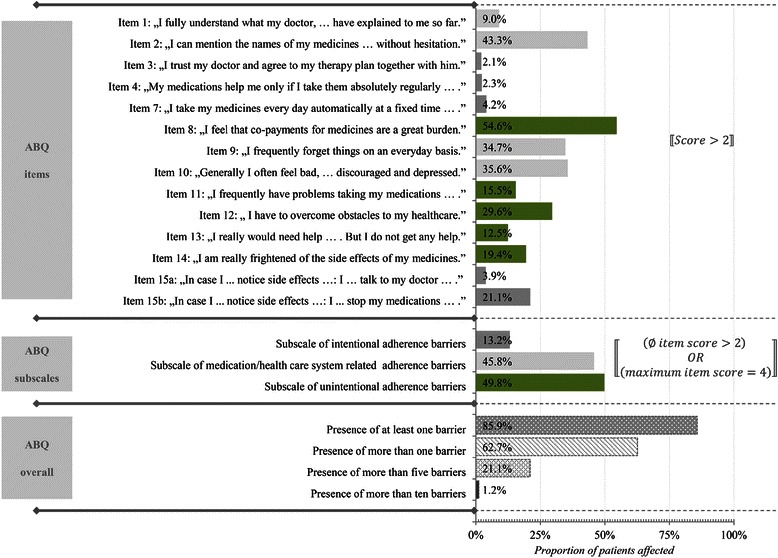
**“Proportion of patients affected by adherence barriers as measured by the ABQ”.** The Figure shows the distribution of the percentage of patients affected by each of the adherence barriers. A patient is defined to be affected by a barrier, if the item score is greater than 2. Furthermore, the proportion of patients which could be assigned to the defined groups barriers’ groups/subscales is shown. A patient was assigned to a barriers’ group if the average score per item belonging to this subscale was greater than 2, or at least one subscale item had a score of 4.

The observed overall ABQ score based on the reduced ABQ-scale (14 items; score of 1–4 per item) ranged from 14 to 52 with a mean of 25.35 (SD = 6.46). A range of 5 to 9 was observed with regards to the scores of the five-item intentional adherence barriers subscale (mean = 7.00, SD = 2.15). With regards to the five-item medication- or health care system-related adherence barriers subscale and the four-item unintentional adherence barriers subscale, scores from 5 to 20 (mean = 9.89, SD = 3.41) and from 4 to 16 (mean = 8.47, SD = 2.57) were observed, respectively. Figure 1 shows the proportion of patients who were affected by the individual barriers (assuming a barrier existed in case of an item score of at least 3).

Figure 1 also shows which percentage of patients could be assigned to the defined groups barriers’ groups/subscales. A patient was assigned to a barriers’ group if the average score per item belonging to this subscale was > 2, or at least one subscale item had a score of 4. In 85.9% of the patients, there was at least one adherence barrier present. More than five barriers were present in 21.1% of the patients. The most commonly mentioned adherence barrier was the feeling that co-payments were a great burden. Based on the three subscales, the following were present: in 13.2% of the patients, intentional adherence barriers; in 45.8% of patients, medication/health care system-related adherence barriers; and in 49.8% of the patients, unintentional adherence barriers.

### External validity of the ABQ and tendency towards social desirability

The overall scores related to the reduced 14-item ABQ and its subscales correlated significantly with the chosen validation variables in both external validation analyses (Table 5). The ABQ score had a stronger correlation with the modified ARMS score (self-report to identify the extent of NA) than with the TTR. However, the correlations with the TTR are still significant for all scales of the ABQ, and show that an increase in number/strength of adherence barriers is associated with a decrease in the TTR, indicating a poorer quality of anticoagulation quality.

**Table 5:** **Spearman rank correlation between ABQ (and subscales), the modified AMRS score and the TTR**

	Modified adherence to refills and medications scale (ARMS)	Time in therapeutic range (TTR)
	N = 401	N = 371
**ABQ**	0.438*	- 0.161***
**Subscale 1 (intentional barriers)**	0.430*	- 0.127***
**Subscale 2 (medication-/health care system-related barriers)**	0.326*	- 0.156**
**Subscale 3 (unintentional barriers)**	0.317*	- 0.103***

*p < 0.001 (2-tailed).

**p < 0.01 (2-tailed).

***p < 0.05 (2-tailed).

ABQ: Adherence Barriers Questionnaire, ARMS: Adherence to Refills and Medications scale, TTR: Time in Therapeutic Range.

Of the 371 patients with available information on TTR, 35.0% had a poor quality of oral anticoagulation (TTR < 60%). Patients with a high ABQ score (above the median of 25), which indicates an increased number of existing adherence barriers and/or a high strength of these barriers, were significantly (p < 0.005, Pearson Chi-Square) more likely to have a poor anticoagulation quality than patients with a low ABQ score (44.6% versus 27.3%).

Based on the additional five questions with regards to social desirability, a subgroup of 322 patients with no strong tendency of responding behaviour towards social desirability (social desirability score < 5) was identified. For this subgroup of patients, the ABQ scores (reduced 14-item ABQ), as well as scores related to its subscales, showed a stronger correlation to the modified ARMS score on a remaining high level of significance (Table 6). Also, the correlates with the TTR increased, but for the third subscale of unintentional adherence barriers the correlation was on an insignificant level. In this subgroup of patients without a tendency towards socially desirable responding behaviour, patients with an overall ABQ score of > 25 were significantly more likely to experience poor anticoagulation quality (43.8% versus 24.7% affected patients with n = 275 patients, p < 0.005).

**Table 6:** **Spearman rank correlation among the ABQ (and subscales), the AMRS score and the TTR for the subgroup of patients without a high probability of social desirability bias**

	Modified adherence to refills and medications scale (ARMS)	Time in therapeutic range (TTR)
	N = 298^+^	N = 275^÷^
**ABQ**	0.468*	- 0.189**
**Subscale 1 (intentional barriers)**	0.435*	- 0.197**
**Subscale 2 (medication-/health care system-related barriers)**	0.373*	- 0.174**
**Subscale 3 (unintentional barriers)**	0.332*	- 0.101***

^+^Subgroup of patients with a social desirability score < 5 and with complete ABQ as well as modified ARMS data.

^÷^Subgroup of patients with a social desirability score < 5 and with complete ABQ, who received vitamin K antagonist and had at least 2 INR values available.

*p < 0.001 (2-tailed).

**p < 0.005 (2-tailed).

***p = 0.094 (2-tailed).

ABQ: Adherence Barriers Questionnaire, ARMS: Adherence to Refills and Medications scale, TTR: Time in Therapeutic Range, INR: International Normalized Ratio.

## Discussion

The psychometric analyses conducted present a high reliability and criterion-related validity of the developed ABQ. The factor structure obtained for the different subscales supports previous results of the adherence barriers research in showing the importance of three different subscales [3,5,46]. The first group/subscale describes intentional adherence barriers. In case these barriers are present, which we assumed if this subscale shows an average score of > 2 or one item of this subscale has a score of 4, a patient, because of his attitudes or negative beliefs, consciously decides to deviate from the treatment plan. On the other hand, there are unintentional adherence barriers, like forgetfulness, depression, or lack of knowledge, which belong to the second ABQ subscale. The third subscale addresses factors like co-payments, missing help/support or special properties of the drugs, and was labelled to be the subscale describing medication- or health care system-related barriers.

All subscales revealed good internal reliability as well as high validity through demonstrating significant correlations with the used adherence self-report instrument (modified ARMS) and the clinical outcome TTR. So, the ABQ can be used as a tool to identify any adherence barriers that may be present in a patient; in this case, it is used on an item-specific basis. Furthermore, the presence of certain adherence barriers groups as defined by our three subscales can be identified. Thus, the ABQ enables scientists as well as clinical practitioners to align certain adherence interventions to specific adherence barriers that may be present in specific patients.

### Limitations

We acknowledge some limitations of our analysis. First, because of data limitations, we were only able to use AF patients treated by GPs for our validation. This may limit generalizability to other settings. Particularly, patients with AF tend to be older and more morbid. Therefore, further research is needed to show the effectiveness of the questionnaire in other settings. Nevertheless, the analysed patient samples were characterised by a high average rate of medication dependency and occurrence of chronic diseases. Second, we used the ABQ in a German treatment setting only and there probably exist country specific adherence barriers (e. g. out-of-pocket cost). It needs to be seen whether the ABQ shows similar validity and consistency when applied in other countries. Third, our survey design did not facilitate the assessment of test-retest reliability that should be proven in further investigations. Fourth, 138 out of 570 patients (24.2%) did not complete the ABQ. The characteristics of these patients do not differ from those of all ACT-AF patients or from those of the patients who completed the ABQ without any gap (Table 1). However, most of these patients (n = 76) did not complete the other parts of the survey as well so that non-response to the ABQ items seems not to be an ABQ-specific phenomenon. Of those patients who did complete the other questions of the survey but could not complete the ABQ, only one patient did not answer any of ABQ items, and a large proportion of patients only missed one (67.7%) or two (12.9%) items of the ABQ. Item 15b was the item with the highest number of gaps (35 of 62 patients 56.4%). It is possible that patients consider items 15a and 15b as alternative items and think they finished the questionnaire after answering item 15a. A renumbering of these items should be done and the effect should be proven in further investigations. Fifth, we decided to use self-reported NA and TTR as external validation criteria. So, there might be more objective measures, which are more suitable to validate our tool (e. g. data derived from medication event monitoring systems or laboratory data). However, self-reported NA and TTR were chosen because they seemed to be both objective measures of medication adherence/clinical effectiveness of medication without being influenced by patient behaviour within the setting. The ARMS was involved in a regular survey within the prospective study period and the INR values for calculating the TTR were measured in a regular care setting. Nevertheless, the ABQ correlated stronger with the ARMS than with the TTR, which may be due to several reasons. On the one hand, the TTR is influenced by other factors, like nutrition. On the other hand, it might be that both the ABQ and the ARMS scores are affected by a self-report bias. The data were collected at a scheduled GP appointment. To the extent that appointment keeping indicates compliance with health behaviour, patients who completed the questionnaire may have been more likely to be adherent to medication therapy as well. At the same time the social desirability bias may be higher when patients answer in a medical environment. However, additional questions were included to control for the tendency of patients to response in a socially desirable manner, and separate subgroup analyses were conducted. Our analysis shows that the predictive power of the ABQ improves if socially desirable responding behaviour is excluded. Based on this analysis, future research may explore whether a more sophisticated ABQ item scoring methodology adjusting ABQ scores by “socially desirability scores” improves the efficacy of the ABQ in identifying existing adherence barriers.

## Conclusions

The ABQ is a practical, reliable, and valid instrument for identifying specific barriers to medication-related adherence. The questionnaire has the potential to support the physician-patient communication as well as the implementation of tailored interventions to improve adherence. Future research is required to examine the usefulness of the ABQ in other settings and its ability to identify patient perception/behaviour changes over time, which may be important for the measurement of success of adherence interventions.

## Additional files

Additional file 1:
**Overview – Review of literature regarding factors associated with medication related NA (2008–2010); the table shows the results of the conducted review of the literature in 2008–2010 with regards to reasons of non-adherence.**
Additional file 2:
**Overview – Review of literature regarding factors associated with medication related NA (2011–2013); the table shows the results of the conducted review of the literature in 2011–2013 with regards to reasons of non-adherence.**
Additional file 3:
**Modified “Adherence to Refills and Medications Scale – ARMS”; in the document the used questionnaire to measure self-reported non-adherence can be found.**

## Abbreviations

ABQ: Adherence Barriers Questionnaire
ACT-AF: Ambulant Care and Treatment of Atrial Fibrillation
AF: Atrial fibrillation
ARMS: Adherence to Refills and Medications Scale
GPs: General practitioners
ICD: International Statistical Classification of Diseases
INR: International normalised ratio
NA: Non-adherence
OAC: Oral anticoagulation
SD: Standard Deviation
TTR: Time in therapeutic range
VKA: Vitamin-K-antagonists
WHO: World Health Organization
