# Supplementary material for: Validation of the Adherence Barriers Questionnaire – an instrument for identifying potential risk factors associated with medication-related non-adherence
Source: BMC Health Serv Res. 2015 Apr 10;15:153. doi: 10.1186/s12913-015-0809-0 (PMC4422301; doi:10.1186/s12913-015-0809-0)
Supplement: Additional file 1: — Overview – Review of literature regarding factors associated with medication related NA (2008–2010); the table shows the results of the conducted review of the literature in 2008–2010 with regards to reasons of non-adherence. [file 12913_2015_809_MOESM1_ESM.pdf]

Additional file 1: Overview – Review of literature regarding factors associated with medication related non-adherence (2008-2010)

Author(s)	Year	Sample	Design	Patient related factors						Medication related factors	Influence on NA	Health care system related factors	Influence on NA
				Socio-demographic	Influence on NA	Unintentional	Influence on NA	Intentional	Influence on NA				
Amberbir A et al.	2008	383 patients with HIV/AIDS	Prospective observational study with structured interviews	Social support	Decrease	Depression	Increase	Use of memory aids	Decrease				
						Forgetfulness	Increase	Feeling bad	Increase				
Campos LN et al.	2008	293 patients with HIV/AIDS	Observational study			Severe anxiety / Depression	Increase						
Chambers S et al.	2008	75 patients with SLE	Cross-sectional study with written questionnaires and semi-structured interviews					Conscious decision to not take medication due to side effects	Increase			High cost of/poor access to medications	Increase
								Perception / belief of a low severity of disease	Increase				
								reference to take drugs only when symptomatic	Increase				
Colombi AM et al.	2008	2.052 patients with DM2 (oral antidiabetics)	Investigation based on administrative data									Higher copayments	Increase
Ingersoll KS, Cohen J	2008	patients with chronic diseases	Review							Complex (dosing) regime	Increase		
Jakubowiak WM et al.	2008	1.389 patients with TB	Cross-sectional study with written questionnaires	Female gender	Decrease	Negative emotional state	Increase	Lack of disease awareness (think of themselves as not ill)	Increase				
				Unemployment	Increase			Unawareness regarding treatment duration	Increase				
				Previous detention	Increase			Aversion to medical personnel	Increase				
								No belief in recovery	Increase				
Karamanidou C et al.	2008	Patients with kidney disease (ERSD)	Review	Older age	Decrease	Depression	Increase	Lack of disease knowledge	Increase	Complex (dosing) regime	Increase		
								Lower self-efficacy	Increase				
								Lack of awareness about the importance / necessity of therapy	Increase				
								Fear of side effects	Increase				
Lacey J et al.	2008	Patients with glaucoma	Cross-sectional study with "home-based" semi-structured interviews	Lower level of education	Increase	Forgetfulness	Increase	Lack of motivation	Increase	Application difficulties (especially in drops)	Increase		
								Lack of belief in the efficacy of the drugs	Increase				
								Fear of illness consequences (blindness)	Decrease				
Liang SY et al.	2008	92 cancer patients	Cross-sectional study with written questionnaires					Opioid-taking self-efficacy	Decrease				
Muyingo SK et al.	2008	2.957 patients with HIV/AIDS	Observational study (Pill-Count / Self-Report)	Self-Report: only one regular sexual partner	Decrease					Previous duration of therapy	Increase	Treating center	
Park JH et al.	2008	2.455.193 patients with hypertonia	Investigation based on administrative data	Female gender	Increase							Increasing number of different prescribers	Increase
				Older age	Decrease								
				Region (city to rural and very rural area)	Increase								
Santos Pde M et al.	2008	158 patients with severe asthma	Prospective cohort study	Lower financial resources	Increase					Side effects	Increase	Length distance to the treating center	Increase
Stoehr GP et al.	2008	343 chronically ill patients with an age of ≥ 65 years	Cross-sectional sturvey			Forgetfulness	Increase			Number of taken drugs (> 4 drugs)	Increase		
						Cognitive functional impairment	Increase						

Legend: The table summarized the results of the conducted review analyzing the existing literature with regards to predictors of medication-related non-adherence (2008-2010). The conducted review is based on a systematic database search (MEDLINE) referring to the related topic in German and English literature of the period from January 2008 to March 2013. The following search terms were used: Adherence [AND] factors (281 hits), Adherence [AND] factor (30 hits), Adherence [AND] predictors (148 hits), Adherence [AND] predictor (11 hits), Adherence [AND] causes (8 hits), Adherence [AND] cause (11 hits), Adherence [AND] barriers (107 hits), Adherence [AND] barrier (7 hits). After removal of duplicates using the database ID (PMID) 597 articles were included in the first qualifying examination. Within this first examination, studies analyzing the adherence of children, dealing with the adherence of physicians in terms of recommended guidelines or relating to the non-adherence to therapy in terms of diet plans, exercise / sports programs, smoking cessation programs or other non-medication therapies were excluded. Finally, 105 articles were included in the review.

Additional file 1: Overview – Review of literature regarding factors associated with medication related non-adherence (2008-2010)

Author(s)	Year	Sample	Design	Patient related factors						Medication related factors	Influence on NA	Health care system related factors	Influence on NA
				Socio-demographic	Influence on NA	Unintentional	Influence on NA	Intentional	Influence on NA				
Valeberg BT et al.	2008	174 cancer patients	Cross-sectional study with written questionnaires	Female gender	Increase			Increasing pain and / or increasing perception of pain relief through medication	NA senkend				
Wu JR et al.	2008	134 patients with heart failure	Prospective observational study	Social support	NA senkend								
				Financial status	n. s.								
				Ethnicity	n. s.								
Fransen GA et al.	2009	347 patients with dyspepsia	Prospective observational study	Lower level of education	Increase	Forgetfulness	Increase	Lower self-efficacy	Increase				
Friedman DS et al.	2009	196 patients with glaucoma	Prospective observational study	Younger age (< 50)	Increase	Depression	Increase						
				Older age (> 80)	Increase								
				African-American	Increase								
				Lower income	Increase								
Lee Y et al.	2009	480 patients with DM	Cross-sectional survey									Trust in treating physician	Decrease
Mann DM et al.	2009	151 patients with DM	Cross-sectional survey					Lack of awareness of the chronic nature of the disease	Increase	Application difficulties	Increase		
								Belief, medication must be used only when the glucose value is high	Increase				
								Fear of side effects	Increase				
								Low self-confidence with respect to the control of the DM	Increase				
Mulgund M et al.	2009	111 patients with osteoporosis	Follow-up-study (NA-Self-Report / patient chart review)					Lack of awareness of treatment benefits	Increase	Side effects	Increase	High costs	Increase
Ngoh LN	2009	patients with chronic diseases	Review			Limited Health Literacy	Increase						
Nguyen GC et al.	2009	235 patients with inflammatory bowel disease	Cross-sectional survey	Older age	Decrease							Trust-in-physician	Decrease
				Poorer HrQoL	Increase								
				Black race	Increase								
Nichol MB et al.	2009	5.943 patients with hypertonia	Investigation based on administrative data	African-American	Increase							No Medicare eligibility	Increase
Protopopescu C et al.	2009	1.100 patients with HIV/AIDS	Follow-up-study (written questionnaires / patient chart review)	Older age	Decrease	Depression	Increase						
				Increasing duration since first diagnosis	Increase	alcoholism	Increase						
				Lack of social support	Increase								
Quach PL et al.	2009	547 patients with schizophrenia	Prospective observational study	Lack of family support	Increase	Drug dependence	Increase	Negative attitude to drugs	Increase				
				Older age	Decrease			Lack of belief in the efficacy of the drugs	Increase				
Shah NR et al.	2009	3.200 newly treated patients with hypertonia	Retrospective cohort study	Female gender	Increase					Number of taken drugs	Increase	Copayments	Increase
				Older age	Increase								
				Lower "baseline" blood pressure	Increase								
				Increasing number of comorbidities	Increase								
Shah NR et al.	2009	1.132 newly treated patients with DM	Retrospective cohort study	A1c > 9%	Decrease							Copayments ≥ 10 $	Increase
Tanioka D et al.	2009	363 patients with HCV	Prospective observational study	Older age (≥ 55)	Increase							Increasing center size (number of treated patients)	Decrease
				Female gender	Increase								

Additional file 1: Overview – Review of literature regarding factors associated with medication related non-adherence (2008-2010)

Author(s)	Year	Sample	Design	Patient related factors						Medication related factors	Influence on NA	Health care system related factors	Influence on NA
				Socio-demographic	Influence on NA	Unintentional	Influence on NA	Intentional	Influence on NA				
Treadaway K et al.	2009	708 patients with MS	Prospective observational study (with internet based surveys)			Forgetfulness	Increase			Side effects (specifically injection-related irritations)	Increase		
						Depression	Increase						
Turner BJ et al.	2009	202 patients with hypertonia	Cross-sectional survey (phone interviews)					Lack of disease knowledge	Increase			Deficiencies with respect to doctor-patient-relationship	Increase
Turner AP et al.	2009	89 patients with MS	Follow-up-study with monthly phone interviews			Strength of MS-related physical impairment / disability	Increase	Afraid of needles	Increase	Type of disease modifying therapy	n. s.		
Vawter L et al.	2008	1.432 patients with hypertonia	Cross-sectional survey	Older age	Decrease	Forgetfulness	Increase	Belief that one does not need the medication	Increase	Side effects	Increase	Copayments	Increase
				Lower income	Increase	Mental function impairment	Increase					Lack of insurance coverage	Increase
Chan DC et al.	2010	14.257 patients with first prescription of statin	Investigation based on administrative data	Female gender	Increase								
				Older age	Decrease								
				Lower income	Increase								
Glass TR et al.	2010	6.709 patients with HIV	Follow-up-study with written questionnaires	Older age	Decrease	Depression	Increase			Complex (dosing) regime	Increase	Change of doctor	Increase
				Lower level of education	Increase	Regular alcohol consumption	Increase						
Laforest L et al.	2010	179 patients with COPD	Cross-sectional survey			Forgetfulness	Increase			Poly-pharmacy	Increase		
Novick D et al.	2010	6.731 patients with schizophrenia	Prospective observational study			alcoholism / drug addiction	Increase	Hostility	Increase				
O'Carroll R et al.	2010	180 patients after stroke	Follow-up-study with written questionnaires	Older age	Decrease	Cognitive impairments	Increase	Concerns / doubts regarding medication	Increase				
								Lack of conviction regarding the benefits of therapy	Increase				
Park KA et al.	2010	265 patients with DM	Cross-sectional survey	Lower financial resources	Decrease			Low self-efficacy	Increase				
								Low awareness about the severity of the disease (esp. diabetes complications)	Increase				
Shigemura J et al.	2010	1.151 patients with depression	Internet based cross-sectional survey	Older age	Decrease			Lower satisfaction with the medication	Increase	Increasing daily dosing frequency	Increase	Poor doctor-patient-relationship	Increase
Solomon DH et al.	2010	142 female patients with osteoporosis	1-year follow-up-study with written questionnaires			Regular alcohol consumption	Increase	Anxiety / concerns regarding side effects	Increase				
								Believes that too many drugs must be taken	Increase				
								Low awareness about the severity of the disease	Increase				
								Lack of belief in the efficacy of the drugs	Increase				
Souza-Machado A et al.	2010	160 patients with severe asthma	Follow-up-study	Lower financial resources	Increase					Side effects	Increase	Length distance to the treating center	Increase
										Complex (dosing) regime	Increase		
