# Supplementary material for: Validation of the Adherence Barriers Questionnaire – an instrument for identifying potential risk factors associated with medication-related non-adherence
Source: BMC Health Serv Res. 2015 Apr 10;15:153. doi: 10.1186/s12913-015-0809-0 (PMC4422301; doi:10.1186/s12913-015-0809-0)
Supplement: Additional file 2: — Overview – Review of literature regarding factors associated with medication related NA (2011–2013); the table shows the results of the conducted review of the literature in 2011–2013 with regards to reasons of non-adherence. [file 12913_2015_809_MOESM2_ESM.pdf]

Additional file 2: Overview – Review of literature regarding factors associated with medication related non-adherence (2011-2013)

Author(s)	Year	Sample	Design	Patient related factors						Medication related factors	Influence on NA	Health care system related factors	Influence on NA
				Socio-demographic	Influence on NA	Unintentional	Influence on NA	Intentional	Influence on NA				
Ágh T et al.	2011	170 patients with COPD	Cross-sectional survey	Age	n. s.					Complex (dosing) regime	Increase		
Baltazary G et al.	2011	197 patients with HIV/AIDS and 30 physicians	"Face-to-face"-Interviews	Stigmatization	Increase	Forgetfulness	Increase	Lack of awareness of the disease	Increase	Side effects	Increase	Higher copayments / costs	Increase
Ben-Natan M et al.	2011	207 older patients with long-term medication	Cross-sectional survey									Satisfaction with treating physician	Decrease
Bird GC et al.	2011	Physicians	Cross-sectional survey	Older age	Increase			Lack of disease knowledge<br>Lack of awareness of the need of therapy	Increase<br>Increase			Higher costs	Increase
Borgsteede SD et al.	2011	20 patients with DM2	Semi-structured interviews	Lack of social support	Increase			Experience with the medication combined with a daily routine	Decrease	Complex (dosing) regime	Increase		
Chamroonsawasdi K et al.	2011	230 patients with HIV/AIDS	Cross-sectional survey	Familiarly support	Decrease			Lack of disease knowledge<br>Lack of awareness of disease severity	Increase<br>Increase	Side effects	Increase		
Daley DJ et al.	2011	772 patients with Parkinson	Review	Older age<br>Longer duration of disease<br>Lack of social environment	Decrease<br>Increase<br>Increase	Affective disorder	Increase	Lack of awareness of the disease<br>Lack of disease knowledge	Increase<br>Increase	Complex regime / poly-pharmacy	Increase		
Gellad WF et al.	2011	Older patients with long-term medication	Review			Cognitive impairment	Increase	Lack of disease knowledge	Increase	Side effects<br>poly-pharmacy	Increase<br>Increase	Good Physician-patient-relationship	Decrease
Iversen MD et al.	2011	32 patients with osteoporosis, 11 GPs, 1 nurses	Focus group interviews			Difficulties to remember taking instructions	Increase	Lack of disease knowledge	Increase	Side effects	Increase	dissatisfaction with the doctor /consultation	Increase
James BO et al.	2011	137 patients with mental illness	Cross-sectional study with written questionnaires	Stigmatization	Increase					Complex (dosing) regime	Increase		
Kim JH et al.	2011	204 Warfarin patients	Cross-sectional survey					Lack of disease knowledge<br>Lower self-efficacy	Increase<br>Increase				
Krousel-Wood M et al.	2011	1.965 patients with hypertonia	Cohort Study of Medication Adherence Among Older Adults	Female gender<br>Being married	Decrease<br>Decrease	Depression<br>Stressful life	Increase<br>Increase						
Kyser M et al.	2011	528 patients with HIV/AIDS	Multicenter, prospective cohort study	Ethnicity (nonwhite)<br>Unemployment<br>Duration of disease	Increase<br>Increase<br>Increase	Regular alcohol consumption	Increase						
Mauskop A et al.	2011	Statin-therapy	Review	Comorbidities	Increase					Side effects	Increase	Good guideline adherence of treating physician<br>Higher costs	Decrease<br>Increase
Muntner P et al.	2011	284 patients after PCI (Clopidogrel)	Cross-sectional study with written questionnaires			Difficulty to consult physician	Increase	Not be able to / dare to ask his doctor about instructions	Increase			Higher costs	Increase
Nair KV et al.	2011	2.451 patients with hypertonia (non-adherence)	Phone interviews			Forgetfulness<br>Difficulties to come to pharmacy	Increase<br>Increase					Long distance to the treating center	Increase
Salt E et al.	2011	108 patients with rheumatoid arthritis	Cross-sectional survey	Ethnicity (nonwhite)	Increase					Number of taken drugs	Increase		
Sedjo RL et al.	2011	13.593 patients with breast cancer	Investigation based on administrative data	Older age<br>Number of comorbidities (CCI)	Decrease<br>Increase							Higher copayments	Increase
Valldeoriola F et al.	2011	418 Patients with Parkinson	Cross-sectional survey	Being married<br>Lower income	Decrease<br>Increase	Psychiatric symptoms	Increase	Lack of disease knowledge	Increase				
Wakibi SN et al.	2011	403 patients with HIV/AIDS	"Face-to-face"-Interviews			Forgetfulness	Increase			Complex (dosing) regime	Increase	Long distance to the treating center	Increase

Legend: The table summarized the results of the conducted review analyzing the existing literature with regards to predictors of medication-related non-adherence (2011-2013). The conducted review is based on a systematic database search (MEDLINE) referring to the related topic in German and English literature of the period from January 2008 to March 2013. The following search terms were used: Adherence [AND] factors (281 hits), Adherence [AND] factor (30 hits), Adherence [AND] predictors (148 hits), Adherence [AND] predictor (11 hits), Adherence [AND] causes (8 hits), Adherence [AND] cause (11 hits), Adherence [AND] barriers (107 hits), Adherence [AND] barrier (7 hits). After removal of duplicates using the database ID (PMID) 597 articles were included in the first qualifying examination. Within this first examination, studies analyzing the adherence of children, dealing with the adherence of physicians in terms of recommended guidelines or relating to the non-adherence to therapy in terms of diet plans, exercise / sports programs, smoking cessation programs or other non-medication therapies were excluded. Finally, 105 articles were included in the review.

Additional file 2: Overview – Review of literature regarding factors associated with medication related non-adherence (2011-2013)

Author(s)	Year	Sample	Design	Patient related factors						Medication related factors	Influence on NA	Health care system related factors	Influence on NA
				Socio-demographic	Influence on NA	Unintentional	Influence on NA	Intentional	Influence on NA				
Wasti SP et al.	2011	Patients with HIV/AIDS	Review	Stigmatization	Increase	Forgetfulness	Increase	Lack of self-efficacy	Increase	Side effects	Increase		
				Financial problems	Increase								
				Lack of social support	Increase								
Bailey GR et al.	2012	59 patients with DM	Cross-sectional study with written questionnaires	General poor health	Increase							Higher costs	Increase
				Low severity of the disease	Increase								
Barraco A et al.	2012	650 patients with bipolar disorder	Multicenter, prospective cohort study	Younger age	Increase	Regular alcohol consumption	Increase	Negative attitudes to therapy	Increase				
Chisholm-Burns M et al.	2012	512 patients after kidney transplantation (immunosuppressive)	Cross-sectional survey	Older age (18-29 versus 46-64)	Decrease	Forgetfulness	Increase	Belief in the need of therapy	Decrease				
								General dissatisfaction with life	Increase				
Cui Z et al.	2012	4.660 patients with fibromyalgia	Investigation based on administrative data	Older age	Decrease	Depression	Increase						
				Comorbidities	Increase								
Dessie A et al.	2012	384 patients with hypertonia	Cross-sectional survey	Number of comorbidities	Increase			Lack of disease knowledge	Increase			Long distance to the treating center	Increase
Devold HM et al.	2012	7.610 patients with osteoporosis	Investigation based on administrative data	Older age (40-59 vs. 70-79); only in women	Decrease								
				Higher income; only in women	Decrease								
				Higher level of education, only in men	Decrease								
Efficace F et al.	2012	413 patients with leukemia	Cross-sectional survey	Lack of social support	Increase			Lack of disease knowledge	Increase	Burden of concomitant medication	Increase		
Fedrick F et al.	2012	272 patients with DM	Cross-sectional survey			Regular alcohol consumption	Increase			Side effects	Increase	Long distance to the treating center	Increase
Grigoryan L et al.	2012	176 patients with hypertonia	Secondary analysis of an RCT	Female gender	Increase							Public health care provider	Increase
				African-American	Increase								
Huetsch JC et al.	2012	2.730 patients with COPD	Investigation based on administrative data					Previous adherence to other medication class	Decrease				
Karakurt P et al.	2012	750 patients with hypertonia	Cross-sectional study with written questionnaires	Lack of social environment	Increase	Forgetfulness	Increase	Lack of disease knowledge	Increase			Higher copayments / costs	Increase
						Carelessness	Increase						
Khdour MR et al.	2012	173 patients with COPD	"Face-to-Face"-Interviews and patient chart review	Number of comorbidities	Increase	Depression	Increase	Lack of belief in the efficacy of the drugs	Increase				
								Lower self-efficacy	Increase				
Lamba S et al.	2012	281 patients after kidney transplantation (immunosuppressive)	Cross-sectional study with written questionnaires via Email	Be divorced	Increase	Previous alcoholism	Increase						
						Mental problems	Increase						
Lewis LM	2012	Afro-Americans with hypertonia	Review			Depression	Increase	Lower self-efficacy	Increase			Good doctor-patient communication	Decrease
Lewis LM et al.	2012	253 patients with hypertonia	Secondary analysis of an RCT	Age	n. s.	Depression	Increase	Lower self-efficacy	Increase				
Li WW et al.	2012	200 patients with hypertonia	Cross-sectional survey	Longer duration of disease	Increase			Lower perception of susceptibility to diseases	Increase				
Magura S et al.	2012	131 patients with mental illness (bipolar disorder, depression, schizophrenia)	"Face-to-face"-Interviews	Lack of social support	Increase			Dissatisfaction with medication / therapy	Increase	Side effects	Increase		
								Lower self-efficacy	Increase				
Morales JM et al.	2012	1.983 patients after kidney transplantation (immunosuppressive)	Cross-sectional survey					Perception that one's life is disturbed by the treatment	Increase	Complex (dosing) regime	Increase		
Murata A et al.	2012	151 patients with depression	Cross-sectional survey	Type of depression	-					Side effects	Increase		
O'Neil CR et al.	2012	556 patients with HIV/AIDS	Longitudinal cohort study	Higher age	Decrease	Drug addiction	Increase						
				Female gender	Increase								
				Low income	Increase								

Additional file 2: Overview – Review of literature regarding factors associated with medication related non-adherence (2011-2013)

Author(s)	Year	Sample	Design	Patient related factors						Medication related factors	Influence on NA	Health care system related factors	Influence on NA
				Socio-demographic	Influence on NA	Unintentional	Influence on NA	Intentional	Influence on NA				
Peyrot M et al.	2012	1.530 patients with DM and 1.250 physicians	Internet-based cross-sectional survey of physician and phone interviews with patients			Stress/emotional problems	Increase	Too busy with other things	Increase				
								Skipping meals	Increase				
								Traveling	Increase				
								Embarrassment in the public (injection)	Increase				
Portelli MS et al.	2012	43 patients with HIV/AIDS	Focus group interviews	Stigmatization	Increase							Long distance to the treating center	Increase
Rushworth GF et al.	2012	20 patients after PCI	"Face-to-face"-Interviews					Awareness about the consequences of not taking the medication	Decrease			Good Physician-patient-relationship	Decrease
								Awareness of the treatment benefit	Decrease				
								Fear of side effects	Increase				
Saks EK et al.	2012	160 patients with urinary incontinence	Cross-sectional survey					General belief that drugs are too often used	Increase				
Schoenthaler AM et al.	2012	608 patients with DM and 41 physician	Cross-sectional survey	Longer duration of disease	Increase			Belief in the need of therapy	Decrease	Subcutaneous application (vs. oral)	Increase	Satisfaction with the ability of the physician to explain something	Decrease
Thames AD et al.	2012	181 patients with HIV/AIDS	Follow-up-study	Lack of social support	Increase	Depression	Increase					Satisfaction with the physician	Decrease
Tobi P et al.	2012	701 chronically ill patients	Investigation based on administrative data	Older age	Decrease								
Wasti SP et al.	2012	330 Patients with HIV/AIDS and 34 other "Stakeholder"	Cross-sectional survey and in-depth interviews	Female gender	Increase	Regular alcohol consumption	Increase	Lack of disease knowledge	Increase	Side effects	Increase	Long distance to the treating center	Increase
				Analphabetisms	Increase			Negative attitudes to therapy	Increase				
Wiegand P et al.	2012	88.635 patients with first prescription of Statin	Investigation based on administrative data	Older age (> 75 vs. 45 - 55)	Decrease							Visiting of only one physician	Decrease
				Female gender	Increase							Visiting of only one physician	Decrease
Alison Phillips L et al.	2013	71 patients with hypertonia	Prospective cohort study					"Bad" habits have been influenced by prior therapy experience	Increase				
Bressington D et al.	2013	584 patients with schizophrenia	Cross-sectional survey					Positive attitudes to therapy	Decrease	Side effects	Increase		
								Awareness of the need of therapy	Decrease				
Curkendall SM et al.	2013	Patients with DM2	Investigation based on administrative data	Female gender	Increase					poly-pharmacy	Unclear / variable	Mail-order use	Decrease
				Older age	Decrease								
				Number of comorbidities	Unclear							Lower lever of cost sharing	Decrease
Evon DM et al.	2013	242 patients with HCV	Prospective cohort study	Older age	Decrease	Depression	Increase			Side effects	Increase	Private insurance	Decrease
				Unemployment	Unclear / variable								
				African-American	Increase								
				Unmarried	Increase								
				Low level of education	Increase								
Girotto E et al.	2013	385 patients with hypertonia	Cross-sectional study	Older age (50 - 79)	Decrease	Regular alcohol consumption	Increase					At least one physician consultation per year	Decrease
				Previous heart attack	Decrease								
Gonzalez A et al.	2013	121 patients with HIV/AIDS	Cross-sectional study with written questionnaires			Drug addiction	Increase						
Hanif H et al.	2013	632 patients with HIV/AIDS	Cross-sectional study with written questionnaires	Female gender	Increase								
				Great social support	Decrease								
				Higher Asset Index	Decrease								

Additional file 2: Overview – Review of literature regarding factors associated with medication related non-adherence (2011-2013)

Author(s)	Year	Sample	Design	Patient related factors						Medication related factors	Influence on NA	Health care system related factors	Influence on NA
				Socio-demographic	Influence on NA	Unintentional	Influence on NA	Intentional	Influence on NA				
Jónsdóttir H et al.	2013	255 patients with bipolar disorder	Cross-sectional survey	Higher IQ	Increase	Drug addiction / Alcoholism	Increase	Lack of insight	Increase	Side effects	Increase		
Lang K et al.	2013	28.238 patients with schizophrenia	Investigation based on administrative data			Regular alcohol consumption	Increase	Awareness about the transmission of the disease	Decrease	Previous NA (within "Baseline"-period)	Increase		
Méda ZC et al.	2013	1.043 patients with HIV/AIDS or/and TB	Cross-sectional study with written questionnaires	Higher number of persons in household	Increase			Positive attitudes	Decrease			Poor access to health care for financial reasons	Increase
				Stigmatization (HIV)	Increase								
Naar-King S et al.	2013	158 patients with HIV/AIDS	Prospective cohort study	Lack of social support	Increase			Awareness of the status of disease				Poor doctor-patient relationship	Increase
				African-American	Increase								
Pachi A et al.	2013	Patients with HIV/AIDS	Review			Depression	Increase						
Park YH et al.	2013	241 patients with hypertonia	"Face-to-face"-Interviews			Forgetfulness	Increase						
Tang L et al.	2013	759 patients with coronary artery disease	Investigation based on administrative data	Female gender	Increase								
				Hispanic/Black	Increase								
