# Supplementary material for: Validation of the Adherence Barriers Questionnaire – an instrument for identifying potential risk factors associated with medication-related non-adherence
Source: BMC Health Serv Res. 2015 Apr 10;15:153. doi: 10.1186/s12913-015-0809-0 (PMC4422301; doi:10.1186/s12913-015-0809-0)
Supplement: Additional file 3: — Modified “Adherence to Refills and Medications Scale – ARMS”; in the document the used questionnaire to measure self-reported non-adherence can be found. [file 12913_2015_809_MOESM3_ESM.pdf]

Additional file 3: Modified “Adherence to Refills and Medications Scale – ARMS”

	All of the time	Most of the time	Some of the time	None of the time
How often do you forget to take your medicine?	☐	☐	☐	☐
How often do you decide not to take your medicine?	☐	☐	☐	☐
How often do you forget to get prescriptions filled?	☐	☐	☐	☐
How often do you run out of medicine?	☐	☐	☐	☐
How often do you skip a dose of your medicine before you go to the doctor?	☐	☐	☐	☐
How often do you miss taking you medicine when you feel better?	☐	☐	☐	☐
How often do you miss taking you medicine when you feel sick?	☐	☐	☐	☐
How often do you change the dose of your medicines to suit your needs (like when you take more or less pill than you’re supposed to)?	☐	☐	☐	☐
How often do you forget to take your medicine when you are supposed to take it more than once a day?	☐	☐	☐	☐
How often do you plan ahead and refill your medicines before they run out?	☐	☐	☐	☐
